# Human prion diseases and the prion protein – what is the current state of knowledge?

**DOI:** 10.1515/tnsci-2022-0315

**Published:** 2023-10-16

**Authors:** Reinhold Nafe, Christophe T. Arendt, Elke Hattingen

**Affiliations:** Department of Neuroradiology, Clinics of Johann Wolfgang-Goethe University, Schleusenweg 2-16, 60528 Frankfurt am Main, Germany

**Keywords:** Creutzfeldt–Jakob disease, variably protease-sensitive prionopathy, fatal familial insomnia, Gerstmann–Sträussler–Scheinker disease, Kuru

## Abstract

Prion diseases and the prion protein are only partially understood so far in many aspects. This explains the continued research on this topic, calling for an overview on the current state of knowledge. The main objective of the present review article is to provide a comprehensive up-to-date presentation of all major features of human prion diseases bridging the gap between basic research and clinical aspects. Starting with the prion protein, current insights concerning its physiological functions and the process of pathological conversion will be highlighted. Diagnostic, molecular, and clinical aspects of all human prion diseases will be discussed, including information concerning rare diseases like prion-associated amyloidoses and Huntington disease-like 1, as well as the question about a potential human threat due to the transmission of prions from prion diseases of other species such as chronic wasting disease. Finally, recent attempts to develop future therapeutic strategies will be addressed.

## 1 Introduction

The chronological order of the history of human prion diseases begins with the description of patients with a previously unknown brain disease by Hans Gerhard Creutzfeldt and Alfons Maria Jakob in the year 1920, both independent from each other. In 1913, the first patient was examined in Wroclow (called Breslau, at this point in time) by Creutzfeldt who described speech abnormalities, confusion, and myoclonus [1]. In 1951, Australian patrol officers met an unknown disease among the so-called Fore people in the eastern of Papua New Guinea. The disease name was given by the characteristic trembling of the patients, which derives from the word “Kuru” of the Fore language [2]. Because of neuropathological similarities between the disease scrapie in sheep and Kuru and Creutzfeldt–Jakob disease in humans, a common mode of infection was postulated, leading to the term “slow virus diseases.” Still in the year 1973, these diseases were thought to be “caused by filterable viruses with strange physical and biological properties” [3]. Already in the year 1967, however, John Stanley Griffith formulated the hypothesis that the agent causing the disease scrapie in sheep is “probably a protein without nucleic acid” [4]. This hypothesis can be considered the first formulation of the “protein-only hypothesis” which states that the infectious agents are nothing else than misfolded proteins that cause prion diseases like scrapie. Stanley Prusiner and co-workers subsequently supported this hypothesis and investigated the sedimentation behaviour and the chemical properties of the scrapie-agent [5,6]. In 1982, Prusiner described a prion as “a small proteinaceous infectious particle, which is resistant to inactivation by most procedures that modify nucleic acids” [7,8]. This finally led to the replacement of the slow-virus-theory and to the term “prion” derived from “protein” and “infectious.” In 1983, the group of Stanley Prusiner formulated the presumption that the prion contains only one major protein, namely PrP [9]. In 1985, the group confirmed that the molecular and biologic properties of the Creutzfeldt–Jakob agent are sufficiently similar to those of the scrapie prion protein and that Creutzfeldt–Jakob disease should be classified as a prion disease [10]. It is therefore appropriate to begin a review on human prion diseases with the infectious agent PrP^Sc^ itself, since even its physiological form prion protein (PrP^C^) is still a subject of current research, especially concerning its molecular functions.

## 2 The prion protein – physiology and pathophysiology

The gene of the human prion protein (*PRPN* gene) is located on chromosome 20 (20p13) and contains three exons. The open reading frame for the transcription of the PrP mRNA and currently known prion disease-associated mutations and polymorphisms are positioned entirely within exon number 3. For the synthesis of the normal “cellular” PrP^C^, the *PRPN* gene encodes a 253 amino acid precursor protein. The following generation of mature prion protein PrP^C^ with a length of 209 amino acids takes place by the removal of an N-terminal and C-terminal signal peptide (amino acids 1–22 and 232–253) and by the addition of a glycosylphosphatidylinositol (GPI)-anchor at the C-terminal end. The structure of PrP^C^ is well investigated and consists of an N-terminal domain with an octapeptide repeat (OPR) sequence capable of binding Cu^2+^ and Zn^2+^ metal ions, as well as a hydrophobic section and a C-terminal domain containing three α-helices, two ß-strands, and a disulphide bridge between amino acids 179 and 214 [11,12,13]. Of major importance for the clinical classification of prion diseases are two N-linked glycosylation sites at the amino acids 181 and 197 and a methionine/valine (M/V) polymorphism at codon 129. The largest proportion of mature PrP^C^ molecules is attached by the GPI anchor to the outer side of the cell membrane. A small portion of PrP^C^ was observed within the Golgi apparatus and the nucleus; however, PrP^C^ anchored to the cell surface is considered to be the main functional form [11,12,14,15]. Mature PrP^C^ can undergo physiological proteolytic processing by the “α-cleavage” occurring between residues 110 and 111, being the most common process resulting in a soluble N1 fragment and a membrane-anchored C1 fragment. It is considered that this α-cleavage has protective effects due to the resistance of the C1 fragment to misfolding and binding capacities of the N1 fragment to toxic protein assemblies [12,16]. The “ß-cleavage” takes place more close to the N-terminus near the octapeptide region and frequently occurs under pathological conditions such as oxidative stress, although it was also described under physiological circumstances [12,16,17]. A third cleavage event called “shedding” takes place at the C-terminal end, leading to the removal of the GPI-anchor and release of an almost full-length PrP^C^ into the extracellular space mediated by the metalloproteinase ADAM10 (A Disintegrin and Metalloproteinase domain-containing protein 10). So far, ADAM10 seems to be the only sheddase for PrP^C^ and research concerning its role under physiological and pathological conditions is still ongoing [16,18]. The central nervous system (CNS) is the major site of PrP^C^ expression and, besides neurons, PrP^C^ is also expressed in astrocytes, oligodendrocytes, and microglia. In astrocytes, PrP is known to be involved in astrocyte maturation during embryonal development, and together with stress-inducible protein 1, it prevents astrocytes from cell death [19,20]. The presence and function of PrP were also studied in oligodendrocytes and in oligodendrocyte precursor cells (OPCs), and of note, proliferation of OPCs took place even in PrP knockout mice. This proliferation, however, took place more vigorously at the expense of a delay in differentiation, which correlates with changes in the expression of oligodendrocyte lineage markers. According to the authors, this finding suggests that in the absence of PrP^C^, OPCs remain in a more protracted undifferentiated state that may negatively affect the normal course of differentiation to myelinating oligodendrocytes. Therefore, a significant role of PrP^C^ in the maturation of oligodendrocytes and myelin formation within the CNS is addressed [19]. A distinct expression of PrP in microglia has been confirmed in early stages of embryonal development of the human brain. This expression in microglia decreased until the end of gestation together with a steady increase of PrP expression in neurons suggesting a role of PrP in early microglial development [21]. Beside in neurons, expression of PrP was also observed in many non-neuronal cells of the human brain such as choroid plexus cells, ependymal cells, and endothelial cells of brain vessels [12,21]. Even outside the CNS, PrP is expressed in plenty of cell types such as neuronal cells from the peripheral nervous system, in cells of the immune system like lymphocytes and mast cells, as well as in parenchymal cells from many organs such as kidney, liver, intestine, pancreas, and heart [12,19,20,21,22,23].

The physiological functions of the PrP^C^ are still a major topic and so far, only partially understood. Probably, the best-confirmed function of PrP^C^ is its role in the maintenance of peripheral myelin, since PrP^C^ knockout mice developed a demyelinating neuropathy after 60 weeks of age [12,13,24]. There is evidence that the effect of PrP^C^ as a physiological ligand of the G-protein-coupled receptor GPR126 plays a major role in the differentiation of Schwann cells and the maintenance of peripheral myelin [25,26]. Binding properties of PrP^C^ with Cu^2+^ are well known since the late 1990s, but the physiological significance of this interrelationship has not been fully understood up to now. The most likely function is the maintenance of homeostasis for copper, zinc, and iron by PrP^C^ such as the regulation of Cu^2+^ uptake by astrocytes and the synaptic uptake of Zn^2+^ [26,27,28]. A third important function is the involvement in neurite outgrowth and neuronal signalling due to the interaction and modulation of many receptors such as ionotropic AMPA and NMDA glutamate receptors and metabotropic mGluR1 and mGluR5 glutamate receptors [26,29]. One major observation concerning this function is the regulation of glutamate uptake by an interaction between PrP^C^ and the glutamate transporter Excitatory Amino Acid Transporter 3, indicating a role in the protection of neurons from oxidative and glutamate-induced cytotoxicity [30]. A fourth important finding concerning the physiological functions of PrP^C^ was the presence of sleep disturbances in PrP^C^ knockout mice showing lower levels of melatonin, leading to the hypothesis that the loss of functional PrP^C^ is involved in the pathogenesis of fatal familial insomnia (FFI). However, the exact involvement of PrP^C^ in the physiology of the circadian rhythm is unclear up to now [12,31]. Another possible and still enigmatic role of PrP^C^ refers to the immune system, since PrP^C^ is expressed in a variety of cell types such as T-lymphocytes, natural killer cells, macrophages, and mast cells. This interrelationship was studied more intensively under several pathological conditions and a physiological involvement of PrP^C^ in the differentiation of these cells is likely as of yet [12]. Concerning the role of the physiological prion protein PrP^C^ in apoptosis, many studies found evidence that PrP^C^ prevents neurons and even astroglia from apoptotic cell death [20,32,33]. In PrP-knockout mice, increased expression levels of apoptosis-related proteins like Bax, p53, or caspase-3 have been detected, and furthermore, increased Ca^++^ levels in mitochondria were found supporting the view of an anti-apoptotic role of PrP through the caspase-dependent mitochondrial apoptotic pathway [34]. Under pathophysiological circumstances, PrP might show a different role, such as the induction of an increased susceptibility of cells to amyloid ß (Aß)-oligomer toxicity and to apoptosis due to an overexpression of PrP. This in vitro observation additionally showed that the degree of mitochondrial dysfunction, calcium-influx, and cellular oxidative stress was dependent on the degree of PrP expression [35]. Therefore, the current view is supported that PrP^C^ prevents neuronal cells from apoptotic cell death but the role of PrP^C^ concerning apoptosis under pathological conditions needs further investigations, as confirmed by the study of an increased induction of apoptosis and cytotoxicity due to Aß-oligomers in combination with increased expression of PrP [20,32,33,34,35]. Finally, investigations on the physiological functions of PrP^C^ must consider the fact that two other proteins belong to the prion protein family: the protein “Doppel” (Dpl) encoded 20 kBp downstream of the *PRPN* gene (20p13) and the protein “Shadow of Prion Protein” or “Shadoo” (Sho) encoded on chromosome 10 (10q26.3). Several studies have shown that there might be a functional interrelationship between all three proteins PrP^C^, Dpl, and Sho under physiological and pathological conditions. Dpl is expressed in the testis and plays a role in gametogenesis and maintenance of sperm integrity [36,37,38]. Under experimental conditions, Dpl exhibits neurotoxic effects on cerebellar cells which can be blocked by PrP^C^ and Sho [39]. Nevertheless, there is so far no proof of an association between Dpl and the pathogenesis of prion diseases [36,38,40]. Sho is expressed in the CNS and shares neuroprotective properties with PrP^C^, but also counteracting effects of Sho and PrP^C^ have been observed such as Sho-induced drug hypersensitivity in neuroblastoma cell lines being blocked by PrP^C^ [40,41]. Additionally, significantly reduced levels of Sho were found in prion-infected mice [42]. Therefore, the functional interrelationship of the three members of the prion protein family under physiological and pathological conditions should be considered a major topic in future studies. This also applies to a further investigation of the structure of PrP^C^ and its different sites being involved in physiological functions of PrP^C^, the conversion of PrP^C^ to its pathological form PrP^Sc^, and even the role of PrP^C^ under pathological conditions, such as its interaction with oligomers like Aß and the development of neurodegenerative disorders in general. Although the structure of PrP^C^ is well known, its comprehensive functional assessment and discussion depend on further knowledge of the large variety of functions under physiological and pathological conditions, many of which still need to be uncovered and further investigated.

The initial processes that lead to the pathological conversion of normal prion protein PrP^C^ to its pathological form PrP^Sc^ are largely unidentified up to now. This also relates to the question concerning the subcellular site of this conversion, and this question is further complicated by the fact that even the spectrum of physiological functions of PrP and its subcellular localizations are still the subject of intensive research. It is known that not all PrP precursor proteins will enter the endoplasmatic reticulum (ER) and some of them will be retained within the cytoplasm, a process known to be supported by oxidative ER stress [12]. These PrP molecules are able to form aggregates in the cytosol and, although their protein structure is different from PrP^Sc^, they share two features in common with PrP^Sc^ due to partial insolubility and resistance to proteinase K digestion [32]. Under physiological conditions, the nucleus and mitochondria are further important subcellular sites of PrP. Within the nucleus, a role in DNA repair has been supposed for PrP due to its interaction with nuclei acid-binding proteins [32]. Even at the inner mitochondrial membrane, the physiological form of PrP^C^ has been detected, but its exact physiological role regarding mitochondrial function remains elusive up to now [43]. Concerning pathological PrP^Sc^, an electron microscopic study of the hippocampus of infected mice revealed a predominant presence of PrP^Sc^ at invaginations of plasma membranes and at locations with cell-to-cell contact, suggesting that closely apposed membranes may provide a favourable environment for conversion and intercellular spreading of PrP^Sc^ [32]. Even in endosomal and lysosomal compartments, PrP^Sc^ has been detected, as well as free PrP^Sc^ not being associated with membrane-bound intracellular vesicles [44]. On a molecular level, different experimental approaches were undertaken to perform a 3D-reconstruction of PrP^Sc^ using animal models, or even nuclear magnetic resonance techniques and X-ray crystallography for the investigation of PrP^Sc^ from different species including humans [26,45,46]. Performance of a 3D reconstruction of the dimer crystal structure of human PrP^Sc^ provides evidence that dimeric oligomerization of two PrP^C^ molecules may be the initial event in the process of conversion, followed by the formation of several ß-strands arranged parallel or antiparallel to each other [26]. Next, a further conversion of the primary α-helical structure of PrP^C^ into a highly ß-rich structure is supposed to take place. One hypothesis states that this continuous process can be described as an interaction of PrP^C^ and PrP^Sc^ in a “zipper”-like manner in which complementary amino acid side chains from ß-sheets interdigitate with each other and stabilize the growing pathological fibrils [47]. Several attempts were made to calculate and describe the molecular 3D structure of PrP^Sc^, one proposal consisting of a structure with a four-rung ß-solenoid architecture. Using chemical calculation methods and analyses of cryogenic electron microscopy images via Fourier transform methods, a precise distance of 19.2 Å between two repeating monomers and an exact 3D-model of the monomer of mouse PrP^Sc^ could be computed [48,49,50]. However, this is not the only 3D model of PrP^Sc^ and it is likely that the structure of the pathological prion protein differs between species and even between different prion diseases. PrP^Sc^ from clinically ill hamster brains infected with PrP^Sc^ revealed a 3D structure very different from the four-rung ß-solenoid structure described above and showed a more parallel oriented ß-sheet structure with each monomer providing one rung of the ordered fibril core, together with N-linked glycans and glycolipid anchors projecting outward [45]. Another 3D reconstruction of PrP^Sc^ from patients with Gerstmann–Sträussler–Scheinker disease (GSS) has been performed using cryogenic electron microscopy. These filaments consisted of dimeric to tetrameric protofilaments with a previously unseen spiral fold with a thicker outer layer and a thinner inner layer [46]. These different observations on the 3D structure underline the need for a further exploration of the prion protein structure in different species and clinical phenotypes.

Infectious prions can spread into the CNS from extraneural entry sites such as the gastrointestinal tract or lymphoid tissue, where PrP^Sc^ culminates in the germinal centres of lymphoid follicles. Additionally, it is known that lymphoid tissues may serve as a source of new prion strains and it is postulated that lymphoid tissues may be more promiscuous than CNS in replicating prions, although the mechanism of this peripheral replication of prions is unclear [47]. Even a retrograde axonal transport of prions from peripheral nerves and autonomic ganglia into the CNS was experimentally demonstrated [47]. Recent data support the view, that infectious prions utilize axonal networks for peripheral to central transport. Experimental studies revealed that in the case of extraneural peripheral inoculation, prion strains replicated in the lymphoreticular system and spread rostral towards the brain along sympathetic projections and the spinal cord. In the case of intraneural peripheral injections, spread of infectious prions occurred along peripheral axonal pathways to brainstem nuclei and/or cortical areas. This spread of prions is supposed to be consistent with a slow axonal transport mechanism, although the exact speed and the exact subcellular mechanism of this transport are still unknown [44]. Inside the CNS, prions infect neurons and astrocytes and then both cell types contribute to spread the prions resulting in prion-induced neurodegeneration. Exosomes are considered to play a central role in the intercellular trafficking of prions, and also, transport of them through nanotubes was observed [13]. Since PrP^C^ knockout mice are resistant to prion diseases, there is no doubt that a dysfunction of the physiological form of PrP^C^ contributes to the infectious process and to neurodegeneration [13,51]. In vitro and experimental studies revealed different factors contributing to prion-induced neurodegeneration, although the underlying molecular mechanisms remain unknown. Microglia play a dual role in prion diseases because, on the one hand, neuroprotective effects via anti-inflammatory cells and, on the other hand, prion-driven neurodegeneration activated by pro-inflammatory cells were reported [47,52,53,54,55,56,57]. A major feature in prion-infected mice is the upregulation of the unfolded protein response (UPR) causing a phosphorylation of PERK (Protein kinase RNA-like Endoplasmatic Reticulum Kinase) and an inhibition of the translation of synaptic proteins [47,57]. Additionally, a neurotoxic cascade has been described with the activation of NMDA and AMPA receptors and the p38 mitogen-activated protein kinase together with an influx of Ca^2+^; yet, the inhibition of any of these steps resulted in a restoration of the normal morphology of dendritic spines. This observation supports the current view that synapses seem to be the initial anatomical targets of prion neurotoxicity, and NMDA and AMPA glutamate receptors might be the first molecular targets [51,58]. A topic of major importance for the physiological prion protein PrP^C^ is its role as a receptor for different oligomers like α-synuclein and especially Aß, which is supported by the observation that antibodies against PrP^C^ blocked Aß-induced synaptotoxicity in vitro and in mice [13,59]. Even a cascade has been observed in an experimental model showing an activation of the Src kinase Fyn by oligomeric Aß bound to PrP^C^, which in turn leads to the induction of hyperphosphorylation of the tau protein [60]. On the human prion protein, amino acids 23–27 and 95–110 represent the binding sites for oligomeric Aß. Additionally, experimental modification of the region of amino acids 120–144 had influence on the interaction between the N-terminal binding sites of PrP^C^ with Aß, indicating a kind of control function of this region for the binding between oligomeric Aß and PrP^C^ [61,62,63]. Of note, PrP^C^ is also able to bind fibrillary Aß-aggregates or monomeric Aß but with lower affinity compared with oligomeric Aß, which represents the major synaptotoxic conformation of Aß indicating a significant role of Aß-binding to PrP^C^ in the development of Alzheimer-typical pathology [63,64]. This was also addressed in a report from 2005 concerning evidence of an overrepresentation of the M allele at position 129 of PrP^C^ in patients with Alzheimer’s disease (AD) [65]. As a confirmation, a large meta-analysis with more than 4,000 AD patients and more than 4,000 controls showed an increased statistical risk of developing AD for MM-homozygote persons compared with VV-homozygote or MV-heterozygote persons. The authors consider this result as a hint for a significant role of PrP-129 polymorphism to the susceptibility of AD besides the well-known role of APOE-polymorphism [66]. Although the need for further research must be emphasized, all these observations confirm a wide range of physiological functions of PrP^C^, and it is also assumed that PrP^C^ is involved in the development of neurodegenerative diseases like AD.

## 3 Sporadic prion diseases

**Figure 1:**
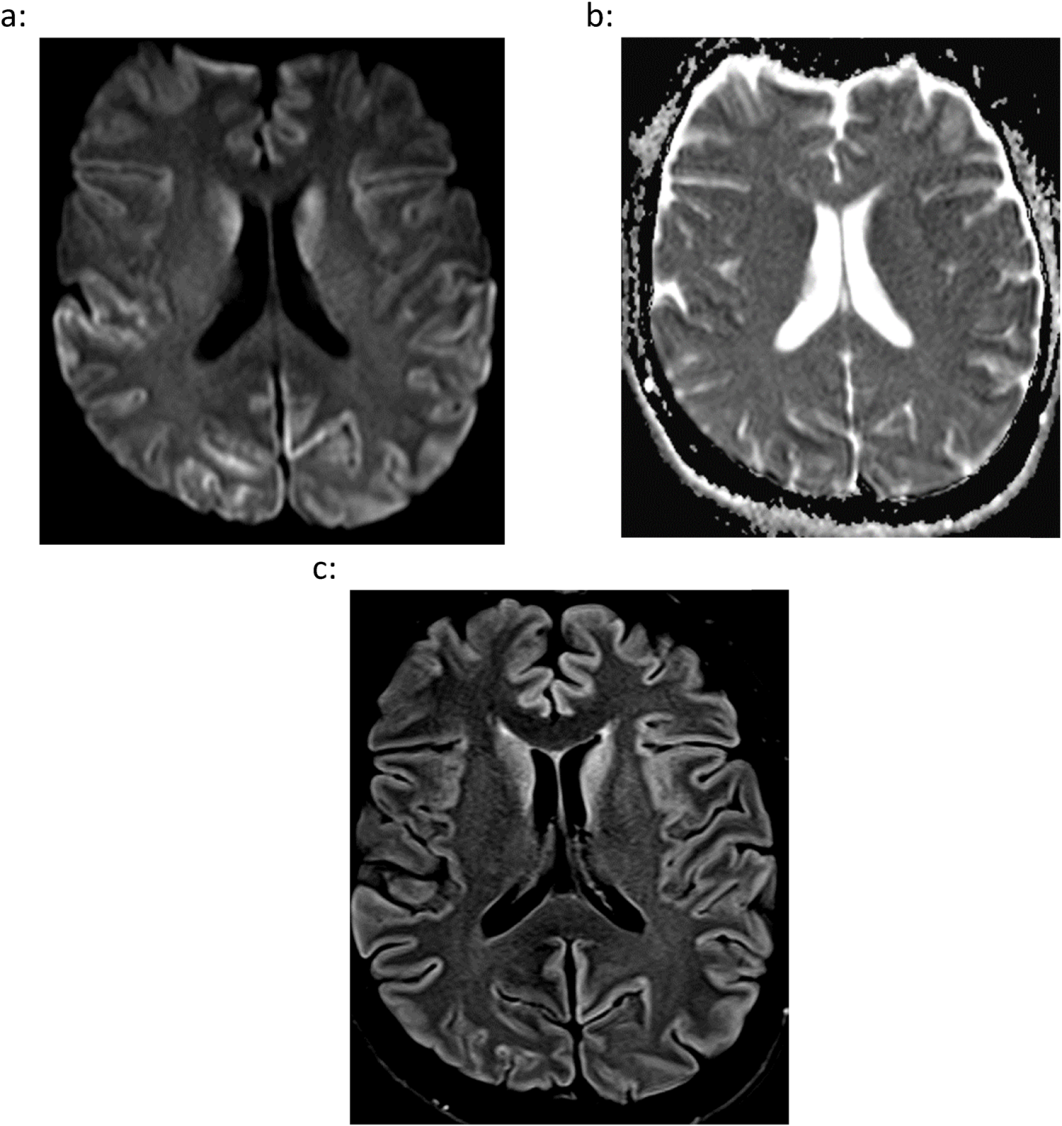
Patient with sCJD (female, 53 years old) initially presenting with progressive cognitive decline leading to akinetic mutism with pyramidal and cerebellar features. No positive family history concerning neurodegenerative diseases, no corneal transplantation. Normal values for glucose, protein, and immunoglobulin G in CSF, normal blood values. Elevation of 14-3-3 protein in CSF and positive reaction in RT-QuIC. MRI showed nearly symmetrical high signals in the parietal, occipital, and temporal cortex and caudate nucleus on diffusion-weighted imaging, DWI (a). The corresponding map for the apparent diffusion coefficient, ADC, presented low values in these regions, which is suggestive of a restriction of the diffusion (b). In the fluid-attenuated inversion recovery sequence, FLAIR, these regions showed a variably hyperintense signal (c). (a) DWI, diffusion-weighted imaging (*b*-value: 1,000 s/mm²), (b) ADC, apparent diffusion coefficient, and (c) FLAIR, fluid-attenuated inversion recovery sequence.

The two sporadic human prion diseases are sporadic Creutzfeldt–Jakob disease (sCJD) and variably protease-sensitive prionopathy (VPSPr). sCJD is the most common human prion disease with a worldwide incidence of 1–2 cases per 1 million people per year. It is assumed that higher incidence rates may be reported in countries with access to established surveillance units [67,68,69]. Age of onset in sCJD shows a peak between 55 and 75 years with a median of 67 years and a median survival time of 5–6 months (Figure 1) [70,71]. The current classification of sCJD mainly depends on the M/V polymorphism at codon 129 of the prion protein. Additionally, the molecular mass of the non-glycosylated fragment of the prion protein after its partial proteinase K-induced digestion is also a part of the classification scheme (Table 1). On western blots, the molecular mass of this non-glycosylated fragment is 21 kDa (type 1) or 19 kDa (type 2), approximately 32 kDa for the mono-glycosylated fragment and 35 kDa for the di-glycosylated fragment. Moreover, cases with a predominance of the non- or mono-glycosylated band (suffix “A”) or of the di-glycosylated band (suffix “B”) can be distinguished [70,72,73]. These sequence and western blot analyses have allowed subclassifying sCJD based on the combinations of the M/V polymorphism (MM, MV, or VV) and the PrP^Sc^ isoform (types 1 and 2). Of note, these sCJD subtypes show differences regarding typical clinical and morphological findings (Table 1). It has become evident that 35% of all patients with sCJD can harbour a mixture of different prion strains resulting in a mixed phenotype, which is not clearly assignable to one of the classical subtypes of sCJD [71,72,74].

**Table 1:** Classification of sporadic Creutzfeldt–Jakob disease (sCJD) according to the polymorphism of codon 129 (MM, MV, VV) and according to the type of the non-glycosylated fragment of PrP^Sc^ within the western blot after partial digestion with proteinase K (“1”: type 1 with a molecular mass of 21 kDa, “2”: type 2 with a molecular mass of 19 kDa)

Subtype (frequency)	Typical clinical findings	Regions typically involved and typical neuropathological findings
MM1 (45–68%)	Rapid progression of dementia, myoclonus, more seldom ataxia, and visual disturbance	Cerebral cortex, basal ganglia, even cerebellum and thalamus can be involved, typical spongiform changes with vacuoles within the gray matter
MM2 (≈10%)	Most commonly as a cortical variant (MM2C) with frequent occurrence of ataxia and myoclonus, dementia with variable course; more seldom as a thalamic variant (MM2T, syn. sporadic fatal insomnia) with insomnia, ataxia, and autonomic dysfunction, clinically indistinguishable from FFI	MM2C: cerebral cortex, basal ganglia, spongiform changes, involvement of the thalamus in a minority of cases; MM2T: thalamus, inferior olive, focal spongiform changes limited to the cortex and limbic region
MV1 (3–9%)	Similar clinical findings as in MM1 subtype with rapidly progressive dementia and myoclonus	Similar to MM1 subtype
MV2 (≈10%)	Ataxia, psychiatric symptoms and extrapyramidal signs, dementia with variable course, typically slower clinical course compared with MM1 and MV1 subtypes	Basal ganglia, cerebellum, variable involvement of cerebral cortex, typically frequent occurrence of amyloid-kuru-plaques in the cerebellum
VV1 (1–4%)	Slowly progressive dementia, psychiatric symptoms with personality changes	Cerebral cortex, basal ganglia, spongiform changes, no involvement of cerebellum
VV2 (10–17%)	Ataxia as the prominent clinical symptom, dementia with late occurrence during the disease	Basal ganglia, thalamus, cerebellum, cortical involvement with spongiform changes in later stages only

Frequency of each molecular subtype as reported from different series of sCJD cases, typical clinical findings, regions typically involved, and typical neuropathological findings [70,71,72,74,84,89].

A major achievement during the last years was the improvement of the Real Time Quaking-induced Conversion Assay (RT-QuIC), showing an optimal specificity of 100% and a high sensitivity of >90% for prion diseases (Table 2) [69,75,76,77]. This is a multi-plate assay with 96-well plates, the reaction mix contains the bio sample with PrP^Sc^ to be detected, recombinant PrP, and thioflavin for the fluorescence-optic detection of the amplified substrate. Therefore, RT-QuIC assay is based on the principle that very few molecules of PrP^Sc^ are sufficient for the amplification of recombinant PrP and thus for a positive result. Several modifications of the RT-QuIC assay including the use of different recombinant PrP molecules have been tested such as chimeric sheep-hamster PrP or even PrP from bank voles. After giving the substrate into the first four plates, incubation starts with intermittent shaking cycles in order to induce fragmentation and to produce new seeds, which are continuously transferred to the next plates. Thioflavin binding of the growing proto-fibrils provides a real-time monitoring of the fluorescence signal, the result of the assay can be obtained after approximately 24 h. In fact, RT-QuIC detects the presence or absence but not the biochemical structure of pathological PrP^Sc^ in the bio sample [75,76,77,78]. The overall sensitivity of RT-QuIC in the diagnosis of human prion diseases is lower than 100%, and this is still due to false negative test results in cases with the infrequent subtypes MM2 or VV1 in sCJD, but also in VPSPr and in the genetic disorders FFI and GSS. By contrast, RT-QuIC shows an almost optimal sensitivity close to 100% for the more frequent sCJD subtypes MM1/MV1, MV2, and VV2. The second important amplification assay for the detection of pathological prion protein is the protein misfolding cyclic amplification (PMCA), which has been first described in the year 2001 [79]. The main difference compared to RT-QuIC is the amplification and even the conversion of a large excess of PrP^C^ induced by PrP^Sc^, so that a continued formation of new PrP^Sc^ will occur. Even for PMCA, different protocols have been tested such as the use of bead techniques or different recombinant prion molecules in order to increase the sensitivity of PMCA for different purposes. In principle, samples are placed in an automatic sonicator that is programmed to perform cycles of incubation, and if necessary, several rounds of PMCA can be carried out to enhance detection by diluting the sample into fresh substrate [80,81]. This test has gained major relevance for the diagnosis of variant CJD (vCJD), which will be described in the section on acquired prion diseases. Especially for sporadic CJD, sensitivity is lower than the sensitivity of RT-QuIC, but ongoing efforts of an improvement have been made leading to the detection of PrP^SC^ in mucosa samples of patients with sCJD with a sensitivity of 79.3% [82]. Although it was not possible to analyse the type of the amplified prion strains from the sCJD patients to a sufficient degree, the principal advantage of PMCA of analysing the amplified prion protein was emphasized. Continued research in order to achieve this goal with sufficient reliability in the future even for peripheral tissue such as oral mucosa has been addressed [82].

**Table 2:** Human Prion Protein and Prion Diseases – Major new Findings and Achievements during the last Years

Human prion protein and prion diseases – new aspects
Prion protein (PrP): – Continuous investigations concerning its physiological functions with a current focus on the maintenance of the homeostasis for metal ions, the involvement of PrP in neurite outgrowth and neuronal signalling, and the process of PrP shedding due to the activity of sheddase ADAM10 [16,26–29,186] – Exploration of the process of pathological conversion to PrP^Sc^ with a focus on the possible role of copper ions, viral infections, dimerization of two PrP molecules [26,28,183–185], and the exploration of the biochemical 3D structure of PrP^Sc^ [49,50,144,145] – Investigation of the downstream effects of PrP^C^ and PrP^Sc^ including effects on the UPR system, on glutamate receptors, Ca^2+^ -influx and activation of p38-MAPK [47,51,57,58]
sCJD: – Improvement of RT-QuIC including the detection of PrP^Sc^ in nasal mucosa and skin specimens with almost 100% sensitivity for the most frequent subtypes [69,75,76,77,86,87] – Exploration of different prion strains with occurrence of more than one prion strain in a high percentage of sCJD cases [71,72,74,75] – Gain of more information concerning the sCJD subtype MM2T (sporadic Fatal Insomnia, sFI) [90,91,92,93]
VPSPr: – Biochemical investigations of the VPSPr prion strain showing typical properties not being present in any other human prion disease such as deficiency of glycosylation and lower seeding activity [99,100,101]
fCJD: – Achievement of an almost 100% sensitivity of the RT-QuIC for cases with frequent mutations (e.g., E200K), still lower sensitivity of RT-QuIC for cases with rarer mutations. Confirmation of pronounced differences between the mutations regarding genetic penetrance [72,115,116,117,118,119,120,121,122]
FFI: – Gain of more detailed clinical information due to a large case series of 131 patients [122] including MRI and PET studies [126,127,128,129]
Gerstmann–Sträussler–Scheinker (GSS) disease: – Confirmation of a distinct clinical and morphological heterogeneity in cases with different mutations and even for cases with the most frequent mutation (P102L), as reported in recent cases series and single case reports [134,135,136,137,138,139]
Variant Creutzfeldt–Jakob disease (vCJD): – Documentation of the last case with initial symptoms appearing in the year 2017 [159] – Technical improvement of protein misfolding cyclic amplification (PMCA) representing the most sensitive test for vCJD [163,164] – Report of the Advisory Committee on the Safety of Blood, Tissues and Organs (SaBTO) in the UK underlining the currently low risk of transmission from blood or platelet products [168]

Due to the 100% specificity and nearly 100% sensitivity of the RT-QuIC assay, a positive result for cerebrospinal fluid (CSF) or other tissue was added to the diagnostic criteria for sCJD according to the National CJD Research & Surveillance Unit (NCJDRSU) in 2017 [83]. Its scheme of the diagnostic procedure in sCJD is a further development of the former World Health Organization diagnostic criteria from 1998, the University of California, San Francisco criteria from 2007 and the magnetic resonance imaging (MRI)-CJD consortium criteria from 2009 [69,71,83,84,85]. All previous diagnostic criteria of prion diseases remain present within the current diagnostic scheme. These include the CSF biomarker 14-3-3, typical electroencephalography (EEG) with periodic sharp-wave complexes, characteristic MRI features such as restricted diffusion on diffusion-weighted imaging (DWI) in neocortical areas and in the region of the basal ganglia (Figure 1), and the clinical symptoms myoclonus, visual and cerebellar disturbances, pyramidal or extrapyramidal signs, akinetic mutism, and progressive cognitive impairment [69,71,77,78]. Additionally, a “probable sCJD” can be diagnosed in case of a positive RT-QuIC result together with a progressive neuropsychiatric or neurological syndrome [69,83]. A further achievement is the high specificity and sensitivity of almost 100% for the detection of PrP^Sc^ by RT-QuIC in specimens from nasal mucosa and skin [86,87]. Investigations of olfactory brushings from patients with sCJD inoculated into transgenic mice show a 10,000-fold lower infectivity compared with the brain tissue and a deficiency of detectable infectivity in CSF samples. According to the authors, this lack of pathogenic infectivity in the RT-QuIC provides evidence that the assay does not replicate biohazardous prions *in vitro* [88]. In case of a negative CSF RT-QuIC result in suspected sCJD, the diagnostic algorithm recommends an analysis of the olfactory mucosa. However, a critical discussion concerning the clinical utility of RT-QuIC utilizing CSF and other tissue is still addressed [69,86].

The classic six subtypes of human sCJD show well-known differences concerning clinical and imaging findings, which can be confirmed by neuropathological post-mortem examinations (Table 1). The two subtypes MM1 and MV1 (45–68 and 3–9% of the cases due to different reports, resp.) exhibit nearly identical clinical features, which can be considered the typical sCJD findings with rapidly progressive dementia, myoclonus and/or visual disturbance and dysphagia. The neocortex is frequently involved in showing restricted diffusion on DWI and spongiform degeneration with immunohistochemical proof of PrP^Sc^ at autopsy. The basal ganglia can also be involved, whereas other grey matter structures such as the thalamus or hippocampus are usually spared [70,89]. The second most common subtype VV2 (10–17% of the cases) shows a more frequent involvement of the basal ganglia, thalamus, and cerebellum with ataxia as the dominant clinical finding. The third most common subtype MV2 (approximately 10% of the cases) can present both dementia and/or ataxia with involvement of all three locations neocortex, basal ganglia, and thalamus and a more slowly progressive clinical course compared with the MM1/MV1 subtypes. The hockey stick sign, referring to bilateral MRI signal hyperintensities on FLAIR (fluid-attenuated inversion recovery) images located in the pulvinar and dorsomedial nuclei of the thalamus, can be present in the MV2-subtype mimicking vCJD, as explained below [70,89]. The rare subtype MM2T, also known as sporadic fatal insomnia (sFI), is characterized by oculomotor disturbances, cognitive decline, and sleep disturbances being confirmed by the medical history or polysomnography. Thirteen confirmed cases in Europe and twelve cases in the United States of America have been reported during the last years. The mean age of disease onset is 43–46 years with a range between 13 and 80 years. The duration of the disease until death can last between 7 and 96 months [90,91]. Besides family history and genetic analysis, sFI is clinically indistinguishable from FFI. Like the other subtypes of sCJD, sFI shows restricted diffusion on DWI in neocortical areas, basal ganglia and thalami. Notably, not all MRI images in published literature show a clearly visible involvement of the thalami. However, ^18^F-FET PET (18-fluoride-fluoro-ethyl-tyrosin positron emission tomography) and post-mortem neuropathological examinations could demonstrate reduced thalamic metabolism and thalamic atrophy with neuronal loss, astrogliosis, and microgliosis in individual cases, respectively [90,91,92,93]. VPSPr was first described in 2008 and was named based on the typical findings on western blots with a band at 6–7 kDa and several additional bands with a variable sensitivity to the proteinase K-digestion. All genetic subtypes of codon 129 can occur in VPSPr cases, with the V/V polymorphism representing the most frequent type [71,94]. VPSPr affects patients between 50 and 70 years, and its clinical course is reported to be very slow with unspecific initial symptoms like impaired short-term memory and difficulties in walking. Cognitive decline or neuropsychiatric symptoms can occur at a later point in time, and time of survival can last up to 5 years. Yet, a case of a 93-year-old woman with no neurological or cognitive symptoms during lifetime but with an autopsy-proven diagnosis of VPSPr was reported. Particularly, MRI findings congruent with sCJD cases can be present, but MRI can also be unremarkable [71,95,96,97]. Histopathology, by contrast, is quite different in comparison to the subtypes of sCJD. Intermediate-sized vacuoles and small plaque-like deposits are often captured, and additional involvement of the cerebellum is frequent but not a must. Together with the typical appearance in western blots, an autopsy examination is so far the only reliable method to diagnose VPSPr [71,96,98,99,100,101]. Recent advances in investigating the abnormal PrP in VPSPr support the view that it is a distinct prion strain not being found in any other prion disease. A defect in protein glycosylation and a lower prion seeding activity are characteristic changes (Table 2) [99,100,101].

Supplementary to the characteristic MRI findings within the subtypes of sCJD, recent studies showed that imaging biomarkers may represent the earliest diagnostically detectable changes in sCJD, even prior to laboratory findings [102,103]. An exemplary case report of a 63-year-old woman with unspecific perioral dystonia highlights the presence of distinct cortical ribboning on DWI at multiple sites. The CSF RT-QuIC was negative at that time but turned positive 6 months later together with further expansion of the restricted-diffusion pattern in imaging [103]. On initial MRI scans of 101 sCJD patients suffering from suspected sCJD, restricted diffusion was detected in the cortex in 67% of the cases, in basal ganglia in 80% of the cases, and in thalami in 51% of the cases, whereas no case showed an exclusive thalamic involvement [104]. By means of voxel-based morphometry in 22 sCJD patients versus 26 age-matched controls, it was demonstrated that cortical and subcortical grey matter atrophy including the thalami was a constant feature in the patient cohort. Interestingly, brain atrophy was present in each case of sCJD, irrespective of the clinical course either with slow or fast progression [105].

CSF biomarkers remain a constantly important part of the diagnostic tools in prion diseases. Besides RT-QuIC, the only marker with a high specificity of 95.6% and a sensitivity of 97.5% in a cohort of 82 sCJD patients is malate dehydrogenase 1 in combination with total tau-protein [106]. Other biomarkers like the 14-3-3 protein, neurofilament light chain protein (NfL), and α-synuclein also show a high sensitivity up to the 9th percentile, but these proteins lack specificity for prion diseases and also might not show elevated levels during the disease course [71,107,108]. Nevertheless, the 14-3-3 protein is included in the current NCJDRSU diagnostic criteria of sCJD [69,109]. Serological biomarkers such as plasma S100b, plasma total tau, and plasma NfL are currently under investigation; however, their diagnostic accuracy is nowadays still lower compared with the CSF biomarkers (Tables 2 and 3) [69,110].

**Table 3:** Diagnostic significance of biomarkers for human prion diseases [70,72,76,77,78,101,106,116,120,121,124,163,164,165]

MRI (FLAIR + DWI)	Specific imaging features for the diagnosis of a prion disease are hyperintensities/restricted diffusion in basal ganglia and/or different regions of the cerebral cortex, even the thalami (esp. pulvinar and dorsomedial nuclei; hockey stick sign) and cerebellum can be involved. No specific imaging features for the differential diagnosis among the prion diseases, including VPSPr, FFI, GSS disease, vCJD, except the typical hockey stick sign in vCJD
CSF (standard analysis)	Important part of the differential diagnostic process: typically, normal values of standard parameters like glucose or protein concentration in prion diseases
CSF (14-3-3 protein)	High sensitivity in sCJD and fCJD but only weak sensitivity of 14-3-3 protein for other prion diseases like FFI or GSS disease; not specific for prion diseases in general, since elevated levels can occur in any other cause of pronounced neuronal degeneration
CSF (other proteins)	α-Synuclein and total tau with remarkable sensitivity but not specific; reports about a considerable specificity of malate dehydrogenase 1 in sCJD and of NfL chain in FFI
RT-QuIC	High sensitivity for the most frequent subtypes of sCJD (MM1, MV1, VV2, MV2) and fCJD with the most frequent mutations like E200K and E196K; limited sensitivity for other subtypes of sCJD, for fCJD cases with other mutations, and for other prion diseases
PMCA	High sensitivity for vCJD; variable sensitivity for other prion diseases
EEG	Typical sharp-wave complexes appear in most sCJD and in fCJD cases, but they are not typical for other prion diseases
Genetic analysis	Analysis of codon 129 polymorphism and search for mutations should be performed for every patient with suspicion of a prion disease; genetic analysis is a prerequisite to diagnose fCJD, FFI, PrP-A including GSS disease, and HDL1
Tonsillar biopsy	Should be performed in cases suspicious for vCJD, but no indication for routine diagnosis of prion diseases

## 4 Genetic prion diseases

**Figure 2:**
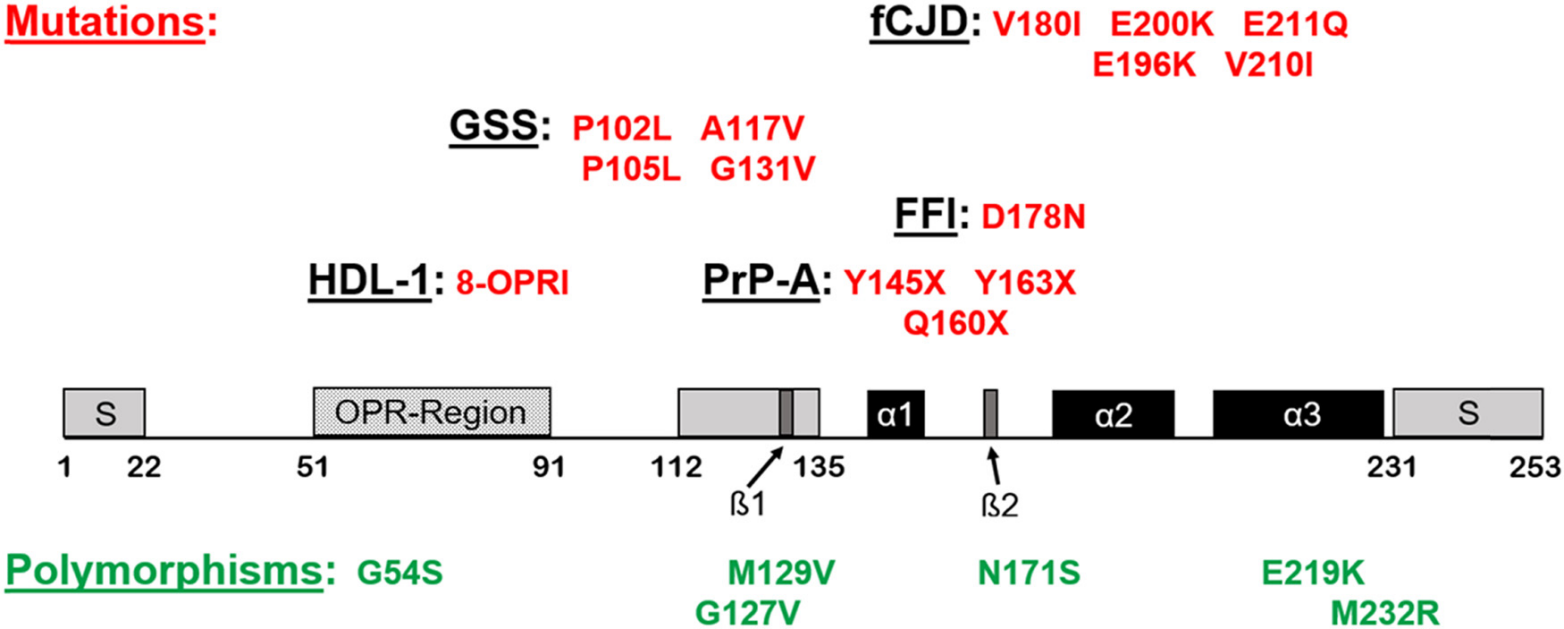
Important mutations of genetic prion diseases, the linear structure and polymorphisms of the human prion protein (PrP): human PrP consists of 253 amino acids with two signal peptides (S) at the N-terminal end (1–22) and the C-terminal end (231–253), an OPR region (51–91), a hydrophobic region (112–135), three α-helical and two short ß-helical sequences. Except for FFI with only one known mutation at codon 178, there are many mutations for each genetic human prion disease being widespread between the N-terminal and the C-terminal region. Nevertheless, the most frequent mutations in familial Creutzfeldt–Jakob disease (fCJD) are located within the C-terminal half of the PrP [111,112,121] and the most frequent mutations in Gerstmann–Sträussler–Scheinker (GSS) disease between the OPR and the hydrophobic region [11,70,111,113]. Most mutations in PrP-A are located within the C-terminal half of the *PRPN* gene [72,131], and HDL1 described in several members of a Swedish family was caused by eight OPR insertions (8-OPRI) within the OPR region [114]. Mutations in the octapeptide region are not only found in patients with the HDL1 phenotype. They were also detected in patients with the CJD and GSS phenotypes [144,145].

The group of genetic human prion diseases includes familial CJD (fCJD), FFI, PrP amyloidoses (PrP-A), GSS, and Huntington disease-like 1 (HDL1). A total of 10–15% of all cases with diagnosed prion disease are inherited due to autosomal dominant mutations of the *PRPN* gene (Figure 2) [70,111,112,113,114]. The most frequent form is fCJD (especially with E200K mutation) followed by the rarer FFI (D178N mutation) and GSS disease (especially with P102L mutation). Importantly, many patients do not show a positive family history, and different reasons have been discussed, including incomplete penetrance or de novo mutations. Therefore, genetic testing is advised for every single case with suspected prion disease (Tables 2 and 3) [115,116]. The prevalence of pathogenic mutations for prion diseases is difficult to ascertain for two reasons. First, the incomplete penetrance of many mutations indicates that their prevalence and thus the number of clinically unaffected persons are higher than expected. Mutations P102L, A117V, D178N, and E200K have a nearly 100% penetrance, whereas other mutations can show a penetrance of less than 1% as addressed below. Second, it is well known that some mutations show differences regarding the geographical origin of the patients. Examples are the mutations V210I and V180I being more frequent in Italy, but also in Japan [115,117]. Another example is T188K mutation being very frequent in China representing nearly 30% of all mutations leading to familial Creutzfeldt–Jakob disease, but this mutation is extremely rare in the western countries [118]. A good estimate of the relative prevalence of all mutations relevant to prion diseases has been provided by a graphical representation showing the relative prevalence of 10,460 patients with genetic prion diseases from 9 countries around the world. As expected, mutation E200K is the most frequent one occurring in the large majority of cases with fCJD. Mutations P102L (GSS) and D178N (FFI) are more seldom as the E200K mutation and represent the second and the third most frequent mutation, followed by mutations in the octapeptide region [115]. All other mutations show a considerably lower prevalence, one example is mutation V210I, which shows a lifetime risk of 10% to develop the phenotype of fCJD [115,117,119]. The clinical presentation of patients with fCJD is largely indistinguishable from patients with sCJD and, furthermore, clinical and pathological variability of fCJD largely depends on codon 129 polymorphism in the same manner as for sCJD. Patients suffering from fCJD with the most frequent subtype MM1/MV1 clinically show a rapid progressive dementia together with the typical histopathology of spongiform changes mainly affecting the cerebral cortex, but also basal ganglia, thalami, and cerebellum to a variable degree. Cases of fCJD with the second most frequent type VV2 present with symptoms of more pronounced ataxia and a more dominant involvement of the deep brain nuclei with less severe involvement of the neocortex [70,71,72,116,120]. Frequently, a younger age of onset in fCJD compared with sCJD is reported; however, this depends on the underlying mutations. For fCJD patients with the most frequent mutation E200K, a median age of onset of 62 years was estimated, being only slightly below the median value of 67 years for sCJD patients [72,115,116]. Sensitivity of CSF biomarkers such as RT-QuIC assay or 14-3-3 protein has been tested in a large series from 302 patients with genetic prion disease including 181 patients with fCJD. Together with patients with other genetic prion diseases, and even 51 healthy controls and 111 patients with other neurological disorders, this study showed a variable sensitivity of the biomarkers dependent on the mutation. Especially for fCJD patients, all mutations showed a considerably high sensitivity in particular for the RT-QuIC assay with 100% sensitivity for cases with mutation E196K, 93% for mutation E200K, and 87% for mutation V210I. Even 14-3-3 protein showed a high sensitivity, but of note, for the most frequent mutations E196K and E200K in fCJD, its sensitivity was only 86 and 82% [121]. As to be expected, even total Tau and α-synuclein revealed a high sensitivity for fCJD, but in contrast to RT-QuIC assay, these CSF markers lack specificity for prion diseases in general [121]. In all other genetic prion diseases, as addressed further below, CSF biomarkers and RT-QuIC assay showed a considerably lower sensitivity, which was also confirmed in other studies [78,121].

FFI is an extremely rare disease with an estimated risk of 1 case per 30 million people. It is characterized by a D178N mutation of the *PRPN* gene combined with M/V polymorphism at codon 129 [71]. Recent advances in understanding the clinical presentation of FFI were made by analysing a large case series of 131 patients that confirmed a broad range of age during disease onset (17–76 years; median age, 48 years; mean survival time, 13 months). The predominant symptoms insomnia and progressive dementia both occurred in more than 80% of the patients. One-third of the patients showed arterial hypertension as an indicator of an autonomic dysfunction [122]. Additional clinical features such as ataxia or myoclonus can overlap with the symptomatology of other prion diseases. Comparison of findings between 128 patients with FFI and 281 patients with other prion diseases revealed additional features useful to distinguish between the prion diseases. Notably, the results of this study indicate that periodic sharp-wave complexes on EEG are not a common feature of FFI in a way that its presence is considered an exclusion criterion [123]. Unlike the high sensitivity of RT-QuIC, 14-3-3 protein, total tau protein, and α-synuclein to diagnose fCJD, all of these markers only revealed low sensitivity to detect FFI. In the above-mentioned study on 302 patients with genetic prion disease, 68 patients with FFI were included and RT-QuIC assay from CSF had 100% specificity but only low sensitivity (28%) to identify FFI patients as patients with genetic prion disease [121]. Unfortunately, in the study with a collective of 131 FFI patients, RT-QuIC was not performed and only 14-3-3 protein was determined as a CSF parameter showing a positive result in 34.5% of the cases [122]. Meanwhile, plasma NfL chain was shown to be a reliable marker to discriminate between 25 FFI cases and 19 controls. Higher serological levels of NfL correlated with shorter survival time from disease onset and shorter time interval between sampling and death [124]. MRI findings in FFI are considered nonspecific, and a diffuse brain atrophy of different degrees can be present. Notably, even cases with a typical clinical course and post-mortem diagnosis of FFI but without any pathological patterns on DWI or in other sequences were reported [125,126,127]. Besides atypical imaging features such as leukoencephalopathy with microbleeds [128], a more typical presentation on MRI and ^18^F-FDG (fluorodeoxyglucose) PET is atrophy and hypometabolism especially of the dorsomedial nuclei of the thalami, respectively [71,129]. Other authors considered the degeneration of the inferior olive nuclei as an additional important characteristic of FFI [126]. Neuropathological examinations corroborated the imaging findings of the thalami revealing severe neuronal loss and gliosis in the dorsomedial nuclei together with the involvement of the pulvinar and anteromedial nuclei. Spongiform changes are not typical for FFI, but they can occur relatively late in cases with a long disease course [71,126,129].

A major advance in understanding genetic forms of prion diseases was made in recent years due to the description of new mutations of the *PRPN* gene in prion protein-amyloidoses (PrP-A) that appear in different organs but with a large variation of the clinical phenotype. Amyloidoses are caused by the accumulation of a PrP^Sc^ fragment with a molecular mass ranging from 6 to 11 kDa, truncated both at the C-terminal and N-terminal ends [72,78]. Many mutations are linked to the phenotype of PrP cerebral amyloid angiopathy (PrP-CAA), and even amyloid deposits in the cerebral or cerebellar cortex or in other brain regions were observed. Spongiform changes can occur in individual cases, indeed the combination of PrP-CAA with numerous tau deposits was identified in the brain of patients with an AD-like symptomatology during lifetime [72,130]. In patients presenting symptoms of chronic diarrhoea, progressive autonomic dysfunction, or peripheral polyneuropathy, widespread deposits of PrP amyloid were found in many organs, sympathetic ganglia, and peripheral nerves [130,131,132]. In individual cases, autopsy revealed PrP amyloid deposits in almost each organ [132]. Overall, various mutations, like stop codon, nonsense, missense, or even truncating mutations, within a large variety of codons were identified [72,131,132,133]. The most prevalent form of genetic PrP-A is GSS. One major recent conclusion highlighted that, on the one hand, the majority of GSS patients showed a typical clinical course and, on the other hand, the very same (and most frequent) P102L mutation in different patients came up with a considerable clinical heterogeneity. GSS disease is characterized by an age at onset of disease in the sixth decade with early onset ataxia and pyramidal and/or extrapyramidal signs, and late-onset dementia accompanied by facultative myoclonus or sleep disturbance. The disease duration of this clinical type has a median value of 50 months [78,108,134]. Although data for codon 129 polymorphism were not available for all retrospectively analysed patients, the presence of methionine was linked to a later onset of disease with a more rapid clinical course, whereas the presence of valine was linked to an earlier onset of around 30 years of age with a long clinical course of more than 10 years [134]. A second and not infrequent clinical type is characterized by a CJD-like symptomatology with early onset dementia and ataxia with a rapid disease progression of less than 10 months [134,135]. Reports about patients suffering from GSS disease and mutations other than those mentioned above confirm the clinically heterogeneous spectrum of that prion disease. One example is the delayed diagnosis in a 40-year-old man with motor symptoms over 2 years and the late development of dementia and neurological symptoms. Postmortem examination confirmed GSS disease due to an A117V mutation and typical PrP-positive multicentric plaques in the neocortex, basal ganglia, cerebellum, and hippocampus [136]. In each of nine GSS cases with P102L mutations, neuropathological examination confirmed the typically small globose or multicentric plaques. In contrast, co-expression of hyperphosphorylated tau, amyloid plaques, or dystrophic neurites was only rarely and focally present in these patients [137]. Imaging findings are consistent with the other prion diseases showing high FLAIR and DWI signals in different cortical regions, basal ganglia, and cerebellum. Cerebellar atrophy can be present, but even cases without any considerable brain atrophy were reported (Tables 2 and 3) [138,139]. Reports about the sensitivity of CSF biomarkers do not reach the numbers of cases with fCJD or even FFI, but in accordance with reports on FFI mentioned above [121], sensitivity of RT-QuIC assay and 14-3-3 protein is much lower for patients with GSS compared with the sensitivity of these markers in fCJD cases. The report on 302 patients with genetic prion diseases included 14 GSS patients with the most frequent mutation P102L, and a sensitivity of 43% was found for RT-QuIC assay and even for 14-3-3 protein [121].

HDL1 was first described in a pedigree of Swedish family members presenting the phenotype of Huntington’s disease but without the otherwise diagnostic trinucleotide repeats in the gene responsible for HD (4p16.3) [140]. Gene defects in HDL1 patients were linked to the *PRPN* gene (20p13) with a characteristic eight OPR sequence [141]. Further phenocopies of HD such as HDL2 with mutations of the JPH3 gene (16q24.2) were not associated with mutations of the *PRPN* gene [142]. The term HDL1 was derived from the clinical presentation of the majority of family members with characteristic chorea, dysarthria, ataxia, and problems with coordination. The age of onset was between 23 and 41 years and disease duration varied between 11 and 23 years. Although not being performed in all cases, neuropathology demonstrated atrophy of basal ganglia, whereas cortical atrophy was present only in focal areas and not in all specimens [140]. Moreover, HDL1 cases showed features not being typical for an HD phenotype, since many patients showed ataxia and psychiatric disturbances as predominant clinical features. Even epileptic seizures, considered to be rare in adult-onset HD, were observed while the typical presentation of chorea was absent in several cases of HDL1. This is in line with other observations underlining that patients with diagnosed HD-like phenocopy often showed atypical clinical features [114,141,143]. Nevertheless, HDL1 must be considered one of the genetically defined HD phenocopies showing a characteristic mutation of the *PRPN* gene [108,114,141]. Of note, mutations in the octapeptide region of the *PRPN* gene are not limited to the phenotype of the disease HDL1. Normally, the octapeptide region of PrP^C^ contains five repeats of 24–27 bp with one nonapeptide and four octapeptide coding sequences, whereas octapeptide mutations in prion diseases can vary with regard to the number of these repeating octapeptide sequences. This explains, in part, that mutations in the octapeptide region can occur in different clinical phenotypes. One example is a mutation with seven OPR insertions in a pedigree with a GSS phenotype [144]. Even mutations in the octapeptide region of PrP from patients with the phenotype of Creutzfeldt–Jakob disease have been found [145].

Besides genetic prion diseases and their corresponding mutations together with the well-known biological significance of codon 129 polymorphism, it must be noted that there are more polymorphisms of the human *PRPN* gene with different effects (Figure 2). G54S seems to be a non-pathogenic allele [146], whereas N171S was described not only in patients with schizoaffective disorders [147,148] but also in an African family with fCJD [149]. The polymorphisms E219K and M232R often occur in Japan and Korea and seem to have a different significance. E219K was reported to possess a protective effect against sCJD [11,150,151], while polymorphism M232R was identified in patients with manifested sCJD but also in healthy persons, leading to the discussion, whether it really represents a polymorphism or a mutation [151,152,153]. Furthermore, G127V is an unequivocally protective polymorphism against an infection with pathological PrP, being addressed in more detail in the following chapter [154,155].

## 5 Acquired prion diseases

Three topics must be subsumed under this heading: the new variant or, put simply, vCJD, iatrogenic CJD (iCJD) as a collective term, as well as Kuru disease, which had occurred endemically in the eastern highland of Papua New Guinea. Variant Creutzfeldt–Jakob disease (vCJD) was first diagnosed in the United Kingdom (UK) in 1996, but its origin is considered to date back up to 1985, when bovine spongiform encephalopathy (BSE) was first described with an increasing annual number of cases in the UK. Since then, more than 184,000 clinically diagnosed cattle with BSE have been documented in the UK together with 5500 cases outside the UK. Since BSE has a long incubation time of 60 months, it has been addressed that even clinically healthy but infected animals could have been used for food supply of animals and humans even before the onset of the epidemic [156,157,158]. Up to the year 2021, 232 patients with variant Creutzfeldt–Jakob disease were reported worldwide, mainly in the UK with 178 cases and in France with 28 cases [73,157]. The last neuropathologically confirmed vCJD case was diagnosed in 2017 in France 7.5 years after an accident of a 24-year-old woman in a laboratory of prion biology. The presence of visual hallucinations and memory impairment as predominant symptoms, a typical hockey stick sign on MRI and post-mortem neuropathological examination confirmed the diagnosis [159]. vCJD affects younger patients than in sCJD with an age of disease onset usually in the third decade of life; however, the oldest patient was 74 years old and the youngest one was an 11-year-old boy from Portugal [157,160]. Duration of the illness is longer compared with sCJD and usually starts with psychiatric symptoms and ataxia. EEG does not show the typical sharp-wave complexes such as in sCJD and the sensitivity of 14-3-3 protein and RT-QuIC are comparably low [108,157]. The two major hints for the intravitam diagnosis of a probable vCJD are the presence of the hockey stick sign on FLAIR and DWI images showing a high specificity and sensitivity, and the accumulation of PrP^Sc^ especially in follicles of lymphoid tissue (e.g., tonsils). Neuropathology typically shows large and confluent florid plaques in cerebral and cerebellar cortex with an eosinophilic amyloid core. Even distinct spongiform changes are present, but especially in the region of the posterior thalamic nuclei, these changes are less prominent and severe neuronal loss and gliosis dominates. It is assumed that this pathology corresponds to the typically high signal observed in FLAIR and DWI images from vCJD-patients within these regions [157,161]. Western blots indicate the subtype 2B, meaning that di-glycosylated prion fragments dominate [108,109,160,161]. All vCJD cases showed the MM polymorphism at codon 129, except one case diagnosed in 2017 that revealed the MV genotype [162]. A major advance in intravitam diagnostic tools for the diagnosis of vCJD was the implementation of an improved procedure of protein misfolding cyclic amplification (PMCA) by independent research groups showing a 100% diagnostic specificity [163,164]. Compared with samples from control patients with other neurological disorders and without prion disease, one study group observed samples from CSF only [163], whereas other study groups examined samples from urine, blood, and CSF with 100% diagnostic specificity regarding all body fluids. The sensitivity of PMCA was only 71.4% in blood samples of the patients with vCJD, but 92.9% in urine, whereas for CSF, a sensitivity of 97.6% has been reported [164,165]. Concerns are still present owing to the significant level of infectious PrP^Sc^ in several, mainly lymphoid tissue, and potential contamination in blood products. In 29,516 samples of appendices removed between 1962 and 1979, indeed seven appendices were positive for PrP^Sc^. One hypothesis is that a significant background level of PrP^Sc^ is present in lymphoid tissue not leading to the clinical phenotype of vCJD, and another one is that human exposure to BSE began much earlier than previously expected [166]. Concerns about blood products are supported by the observation of more than 20 people receiving blood products from donors, who developed vCJD after donation. Three of the recipients developed and died from vCJD between 2003 and 2006. In these cases, the incubation period between blood transfusion and disease onset was 7–9 years [157,160,167]. The advisory committee on the Safety of Blood, Tissues and Organs in the UK established a working group on this topic. The 2021 revised risk assessment shows that the current risk of transmission of PrP^Sc^ from blood or platelet products is predicted to be low (Tables 2 and 3) [168].

Approximately 500 cases with iCJD have been identified worldwide, the first documented one in the year 1972 receiving a corneal transplant [169]. The two largest patient groups suffering from iCJD were 200 recipients of pituitary-derived human growth hormone (hGH) with the majority of cases being treated in 1983–1985 in France and over 200 recipients of dura mater grafts since 1987 with more than 60% of the cases occurring in Japan [107,169,170]. All other causes of iCJD are exceptionally rare, underlined by the description of only two cases with contaminated intracranial EEG electrodes, four cases with contaminated surgical instruments, and ten potential cases with corneal transplantation [169,170,171]. Documentations of clinical findings are largely biased due to the small number of patients and studies predominantly performed on recipients of dura mater grafts. An “intermediate” M strain of 20 kDa was reported in western blots, showing a molecular weight between the non-glycosylated type 1 (21 kDa) and type 2 (19 kDa). This M strain was claimed to represent a reliable criterion to identify cases with iCJD [72], but it has not yet been confirmed in cases others than in contaminated dura mater grafts. Retrospective analysis of 22 patients receiving hGH demonstrated that cerebellar signs with gait ataxia and later development of cognitive decline were the characteristic clinical findings [172]. This is in line with the description of clinical signs in other iCJD cases due to dura mater grafts or even due to contamination of surgical instruments or cornea transplantation [170]. However, distinct differences in incubation times have been observed when comparing iCJD cases with different aetiologies. The mean incubation times for iCJD patients receiving dura mater grafts or hGH were 12 and 17 years resp., with some patients showing an onset of the disease 30–42 years after receipt of dura mater graft or hGH [170]. In contrast, patients treated with contaminated surgical instruments or corneal transplants revealed a mean incubation time of 1–2 years with single patients showing an incidence of up to 27 years after a corneal transplantation [170]. The incidence of iCJD decreased during the last decades and only exceptional cases due to long incubation periods were reported within the last years [72]. However, special attention is addressed concerning the relationship between CJD and a previous corneal transplantation. It is recommended that globally all patients with CJD should be investigated for past corneal transplant history and, if possible, even the cause of death of their corneal donors should be investigated and reported [171]. An important contribution to the validation of diagnostic procedures for patients with iCJD has been made by a retrospective analysis of diagnostic results from 23 iCJD patients including electroencephalogram, neuroimaging results, as well as CSF markers typical for sCJD such as 14-3-3 protein, phosphorylated and total tau, NfL, and RT-QuIC assay. As a result, diagnostic accuracy of all these CSF biomarkers including 14-3-3 protein and RT-QuIC showed the same reliability for the diagnosis of iCJD as for the diagnosis of sCJD [172,173].

The term “kuru” means “tremble” in the language of the Fore people in the eastern highland of Papua New Guinea that represents the endemic region of the prion disease Kuru. The origin of the disease was assigned to the regional endocannibalism, which was first described by Australian patrol officers in the early 1950s. The disease had a typical duration of 1 year starting with progressive ataxia and dysarthria, emotional changes with inappropriate euphoria and laughter, followed by inability to walk and progressive dementia [2,71]. Neuropathology of the disease was investigated in brains of humans and chimpanzees, the latter after disease transmission, showing spongiform changes and vacuoles in the cerebral cortex similar to the findings in scrapie. Most prominent changes, however, were found in the cerebellar cortex with severe moth-eaten vacuolation of Purkinje cells, neuronophagia, widespread astrogliosis and microgliosis, and typical kuru plaques with a pale halo and a diameter of 20–60 µm in cerebral and cerebellar cortex, basal ganglia, and thalami [2,71,174]. A major pathological finding was the presence of pathological prion protein in follicular dendritic cells within the spleen of Kuru victims and this finding is in line with the finding of a widespread presence of PrP^Sc^ in lymphoid tissue from patients with vCJD [2,157]. A long incubation period of up to 50 years was estimated and the last death due to Kuru was documented in the year 2005. As the endocannibalism declined in the late 1950ies, no new case has been described in people born after the year 1959 [175] meaning that Kuru is now considered eradicated [71,174,175]. In line with the significance of PrP 129 polymorphism for patients with sCJD, homozygosity M/M and V/V was overrepresented in Kuru patients, whereas heterozygosity M/V dominated in non-affected Fore-people during the Kuru-epidemic period [2,176]. Importantly, one additional finding in Kuru has an actual significance: G127V polymorphism of the prion protein had a protective effect against Kuru as it was never detected in patients with manifested disease [154]. Recently, monoclonal cell lines expressing the so-called bank vole prion protein (BVPrP) were generated to provide a model for studying the replication of a diverse range of prion strains from different species. A major observation was that cells expressing BVPrP with G127V polymorphism could not be infected with prions. It is assumed that this polymorphism hampers the prion replication cross-species or indeed the spontaneous aggregation [155].

## 6 Discussion

Out of all recent advances in understanding the prion diseases, the technological improvement of RT-QuIC is of upmost importance together with its inclusion into the diagnostic criteria of sCJD [69,73,77]. Remarkable sensitivity was achieved not only in CSF samples but also in nasal mucosa and skin specimens [69,86,87]. It should be noted, however, that RT-QuIC is still not available in every laboratory and that a critical discussion concerning the clinical utility of this technique in CSF and other tissues is still addressed [69]. Additionally, RT-QuIC can be negative in an early clinical stadium of sCJD, when cortical ribboning on MRI is already present [103]. Compared with the almost 100% sensitivity for the most frequent subtypes of sCJD and fCJD, RT-QuIC still showed a limited sensitivity for the rarer subtypes of sCJD, fCJD, vCJD, VPSPr, FFI, and GSS disease [71,78,79,108,121,157]. Besides RT-QuIC, two other biomarkers can be considered specific for prion diseases: malate dehydrogenase 1 with a sensitivity and specificity of over 90% in patients with sCJD [106] and PMCA with an almost 100% specificity and sensitivity to diagnose vCJD [163,164]. All other biomarkers like 14-3-3 protein or CSF tau show variable sensitivity and specificity, but neither of them is specific for prion diseases. Blood biomarkers are being investigated, but their sensitivity is still lower than CSF biomarkers up to now [69,90].

A major clinical finding from large case series is the considerable variability of the phenotype in different human prion diseases, even within the same disease entity or indeed within the same mutations in genetic prion diseases. In conclusion, this explains the considerable overlap of morphological and clinical findings when comparing case series from different prion diseases. Especially, patients suffering from sCJD and fCJD are clinically indistinguishable and, notably, the mean age of disease onset in fCJD with the most frequent mutation E200K is only slightly lower compared with sCJD (62 vs 67 years) [71,115,116]. Indeed, clinical and morphological variabilities were shown to be very pronounced in rarer prion diseases like VPSPr, FFI, and GSS disease, and even cases with definite autoptic diagnosis but without equivocal clinical and morphological findings during lifetime were described [96,125,126,127]. Since 10–15% of all human prion diseases have their origin in a genetic disorder and since many of these patients do not have a positive family history, genetic testing should be performed in each patient with a suspected prion disease. Two disease groups are missing in the majority of review articles on prion diseases, involving the group of genetic PrP-A and HDL1. In PrP-A, GSS disease is the most prevalent disease among others. Many reported cases with PrP-A presented as cerebral amyloid angiopathy, but also a combination with spongiform changes and a CJD-like symptomatology or even with peripheral polyneuropathy, progressive autonomic failure, and chronic diarrhoea occurred [130,131,132]. HDL1 is definitely a phenocopy of Huntington disease (HD) and shows a mutation of the prion protein gene. Thus, it must be considered in any case with suspicion of HD. Importantly, HDL1 does not show the typical trinucleotide repeats on chromosome 4p16.3 in HD [114,141].

The last case with vCJD was reported in the year 2020 in a patient who had an accident in a laboratory of prion biology 7.5 years prior to her diagnosis of vCJD [159]. Another case reported in the year 2017 has gained major interest since it represents the first case with vCJD not being associated with MM but MV type at codon 129 [162]. Consequently, this raised concerns about the possibility of a new vCJD prion strain potentially leading to a new wave of vCJD cases like during the BSE crisis 20 years ago. Up to now, however, there is no hint for that and, together with the low probability of new diagnosed cases due to a prion infection via blood products, risk of transmission is currently considered to be low [168]. The same holds true for other iatrogenic prion diseases, since the large majority were caused by dura mater grafts and the administration of hGH some decades ago, and other causes of iCJD are extremely rare. The cause of Kuru disease was undoubtedly the endemic endocannibalism, which has been successfully terminated since the late 1950s, and in fact, no more case has been observed in people born after the year 1959. Therefore, Kuru disease can be considered eradicated. Current concerns about a threat to human health due to animal prion diseases are very much focussed on chronic wasting disease (CWD) being the most contagious and infectious of all prion diseases. PrP^CWD^ was demonstrated to spread throughout the whole body of the animals including CNS and PNS, muscles, lymphoid tissue, blood, saliva, faeces, and urine [177]. This situation increases the exposure risk to all animal species within that ecosystem and even potentially to humans exposed to CWD-infected meat. The first diagnosis of CWD was made in deer and elk in 1967 in the USA in Colorado and Wyoming. Later, it spread to 29 US states, Canada, and South Korea and, since 2016, Norway, Sweden, and Finland [177,178,179]. Observations that currently argue against a major threat from CWD are the lack of reports about any transmission to livestock or humans in endemic areas and a strong transmission barrier to species other than cervids up to now. The largest human exposure took place in the year 2005 in the state New York with 200 people being unknowingly encountered with CWD-infected meat; however, a 6-year follow-up revealed no cases of human prion disease [177,180]. Another major finding is the lack of evidence for a disease spread from reindeer to lamb or sheep in Norway, as no prions could be detected in gut-associated lymphoid tissue of 425 and 78 animals, respectively [181]. Despite these reports, the coexistence of animals with CWD and humans makes future continuous monitoring for human prion diseases in endemic areas necessary [177,178,179]. For animals, RT-QuIC was modified to provide a high sensitivity and specificity for PrP^CWD^ providing a good basis for a reliable monitoring of this zoonotic prion disease [179,182].

**Figure 3:**
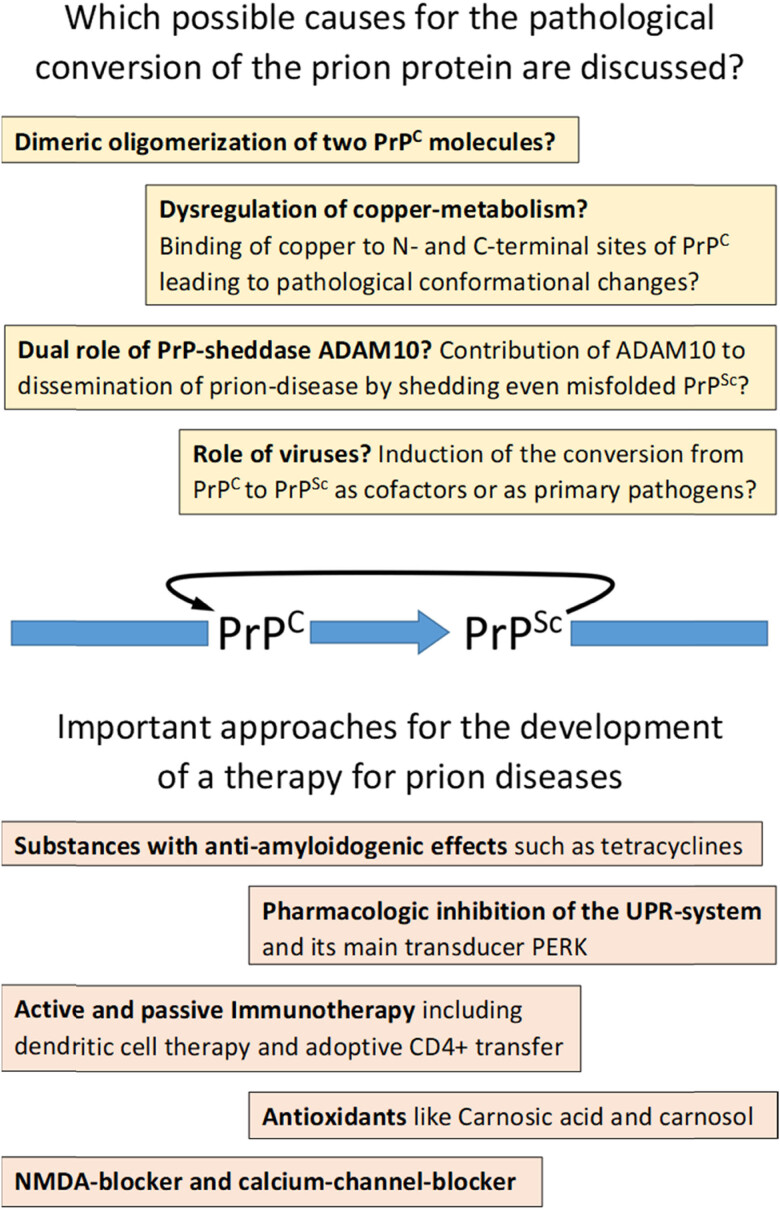
Schematic diagram of the pathological conversion from PrP^C^ to PrP^Sc^ together with its possible causes as discussed above [16,26,28,48–50,183–187]. Former and current attempts of finding appropriate therapeutic approaches representing one of the main goals of prion research [57,189–201].

Advances in understanding the human prion protein were made especially in the field of its physiological functions, the visualization of the conversation process from its physiological to pathological form, and even the discovery of the downstream cascade of prion-induced neurodegeneration. However, causes and early molecular events of the pathological conversion from PrP^C^ to PrP^Sc^ remain poorly understood. Importantly, copper ions binding to the octapeptide region of the N-terminal region of PrP^C^ and interacting with the C-terminal region might play a major role because this interrelationship led to protein kinase K-resistant conformational changes (Figure 3). By contrast, other studies demonstrated a copper-induced inhibition of the conversion process and a delay of disease onset in animals infected with prions [28,183]. An outstanding new finding is the induction of PrP^C^/PrP^Sc^ conversion in neuroblastoma cell lines due to an infection with a neurotropic influenza type A virus (IAV). Moreover, mice infected with these neuroblastoma cells developed a prion disease due to an accumulation of PrP^Sc^ and spongiform degeneration within their brains [184,185]. The authors considered these results a proof of the first non-prion pathogen being involved in the process of prion conversion, and that the infection of neurons with IAV might be a cause or an association factor of sporadic prion diseases [185]. Another important topic is the role of PrP sheddase ADAM10 in prion diseases. Using an antibody specific for the shedded and di-glycosylated version of PrP (sPrP) in a murine model, an inverse correlation between sPrP and PrP^Sc^ levels was observed and interpreted as an interference of sPrP within the conversation process via blocking critical PrP^Sc^ assemblies in the extracellular space [16,186]. By contrast, anchorless and under-glycosylated PrP expressed in transgenic mice were shown to promote prion conversion and aggregation. It cannot be fully ruled out that ADAM10 might contribute to the spread of prions in the brain because, in principle, it is capable to shed even misfolded PrP^Sc^ (Figure 3). Therefore, ADAM10-mediated shedding might play a dual role in prion diseases due to currently unknown factors and cofactors [16,18,187,188]. Up to now, however, protective effects of ADAM10-mediated shedding outweigh, providing a potential target for future therapeutic strategies [16,186].

Attempts on new therapeutic strategies can be classified into three general approaches: lowering the expression of PrP^C^, inhibiting the conversion from sPrP to PrP^Sc^, and blocking the downstream neurotoxic signalling cascade [57,189]. Early attempts to decrease the levels of PrP^Sc^ were made by initially encouraging experimental results using pentosane-polysulphate, doxycycline, quinacrine, and amphotericin B. Yet, no considerable survival benefit or even a reduction of neuropathological changes was observed in humans [189–192]. The UPR is a major regulator in controlling protein structures and oxidative stress induced by the ER, the latter being potentially caused by the accumulation of PrP^Sc^. UPR has gained increasing interest because experimental pharmacological inhibition of its main transducer PERK prevented UPR-mediated translational repression and led to neuroprotective effects within the whole mouse brain [193]. Therefore, pharmacological inhibition of PERK is still considered a potential therapeutic strategy for prion diseases and other neurodegenerative disorders (Figure 3) [189,192,194,195]. Many approaches in experimental immunotherapy with the usage of antibodies against PrP showed significant benefits following passive immunization in mouse models [196,197]. The general and mostly unpredictable problem of antibodies against PrP is their potential neurotoxicity and the insufficient penetrance through the blood–brain barrier (BBB) for many of them. Notably, some antibodies traversing the BBB have been developed in the meantime [189,192,196,197]. A special field of immunotherapy is the dendritic cell therapy and its variant called adoptive transfer of CD4^+^ T cells. A significant delay in disease progression and prion propagation was reported in mice after transferal of CD4^+^ T cells [198,199]. A further success in immunotherapy is the discovery of new pathways to deliver therapeutic antibodies via nanobodies [197]. Out of several strategic attempts to treat prion diseases, two new findings must be highlighted: Carnosic acid and its metabolite carnosol from the kitchen herb rosemary have antioxidant and neuroprotective effects. Tested in a cell model of prion diseases (N2a22L cells), both compounds prevented the formation of PrP aggregates and additionally led to a disruption of already formed aggregates [200]. Since synapses and NMDA receptors belong to the main sites of PrP replication, it has been tested if manipulation of synaptic plasticity could serve as a factor for modulation of PrP^Sc^ formation. In a cell model, combined treatment with an L-type calcium channel blocker and an NMDAR blocker led to a marked reduction of PERK and PrP^Sc^ aggregates [201]. All of these findings reveal novel insights and additional targets to intervene and treat prion diseases in future (Figure 3).

In summary, important new aspects and recent achievements have been presented in our review focussing on human prion diseases and the prion protein. Starting with the continuous investigations of physiology and pathophysiology together with the structure of the prion protein, the improvement of RT-QuIC as a major diagnostic tool has been highlighted together with the diagnostic significance of all other biomarkers and finally, major clinical and molecular aspects of all human prion diseases were explained. A further important aspect is the current lack of evidence for a human threat due to CWD because no single case of a transmission to humans or to animal species other than cervids has been described. Regarding attempts concerning the exploration of potential therapeutic strategies, the broad spectrum of concepts must be emphasized, since prion diseases show a variety of different molecular targets such as the UPR system or NMDA receptors being intensively investigated. Two findings should be given particular attention, namely the protective G127V polymorphism and even the in vitro observation of a disruption of pathological PrP aggregates by the antioxidants carnosic acid and carnosol contained in the kitchen herb rosemary. Besides many other findings, even these observations may harbour the potential of a breakthrough for a successful future therapy of prion diseases, and therefore, even these two findings should be included in the long list of efforts and achievements to open up pathways to a clinically effective therapy for human prion diseases.
